# Harnessing Microbial Volatile Organic Compounds for Crop Protection: Scientific Discovery, Bridging Ecological Function and On‐Farm Application

**DOI:** 10.1111/1751-7915.70313

**Published:** 2026-02-06

**Authors:** Katharina Belt, Lachlan Dow, Marta Gallart, Louise F. Thatcher

**Affiliations:** ^1^ ARC Centre of Excellence in Plant Energy Biology, School of Molecular Sciences University of Western Australia Crawley Western Australia Australia; ^2^ Institute of Agriculture University of Western Australia Crawley Western Australia Australia; ^3^ Commonwealth Scientific and Industrial Research Organisation (CSIRO), Agriculture and Food Acton Australian Capital Territory Australia; ^4^ Commonwealth Scientific and Industrial Research Organisation (CSIRO), Advanced Engineering Biology Future Science Platform Acton Australian Capital Territory Australia

## Abstract

Microbial volatile organic compounds (VOCs) are integral to microbial ecological communication. Their potential as tools for sustainable crop protection is increasingly recognised, yet practical implementation remains limited. There are numerous in vitro lab‐based studies focussed on screening single strains of soil or plant‐associated microbes for their ability to produce VOCs and demonstrate their potential to inhibit plant pathogens or pests. Most of these, however, lack any validation *in planta* or in the field after petri dish experiments. This extends to a lack of understanding on whether the same VOCs are produced in vitro as *in planta*. How do we shift this focus and move from exciting lab‐based discoveries to practical, scalable crop protection solutions for farmers? This opinion piece explores the current state of research on microbial VOCs for crop protection, translational challenges in deploying them on‐farm, and highlights areas where learnings from the ecological roles of microbial VOCs can be leveraged towards field application.

## Graphical abstract

This opinion article highlights how microbial VOCs can support sustainable crop protection and outlines the ecological, analytical and translational challenges that currently limit their field application. It proposes practical and technological pathways to bridge the gap between laboratory discoveries and on‐farm deployment.

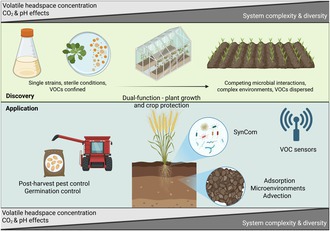

## 1 VOCs As a Biological Alternative in Crop Protection

Agricultural systems are under pressure to reduce reliance on synthetic pesticides, mitigate pest resistance and enhance ecological resilience. Microbial volatile organic compounds (VOCs), characterised by a high vapour pressure, low boiling point and a molecular mass of below 300 Da, offer a biologically inspired alternative. These compounds can act at short or long distances through soil, liquids or air. Microbial VOCs are structurally diverse, and include, for example, alkanes, alkenes, alcohols, aldehydes, ketones, carboxylic acids, esters and lactones, ethers, aromatics (both homo‐ and heterocyclic), terpenes, cyanides and sulphur‐containing VOCs (Bennett and Inamdar 2015; Kemmler et al. 2024; Schenkel et al. 2015; Wang, Ke, et al. 2023). This diversity arises from various biosynthetic pathways, such as primary metabolism, fatty acid synthesis and terpene biosynthesis, leading to both common compounds found across many species, as well as unique compounds specific to certain microbial strains. This is seen in *Actinobacteria*, where strains maintain unique VOC profiles while sharing certain common compounds with related or more distal strains (Choudoir et al. 2019). The specific VOCs produced are influenced by the microbes' genetic makeup, environment (including nutrients and other environmental conditions), and community and/or host dynamics (Ali et al. 2025; Korpi et al. 2009; Morath et al. 2012; Plaszkó et al. 2020).

In nature, VOCs serve as microbial modulators, shaping soil and rhizosphere communities and nutrient cycling, while in association with plants, they can have positive effects on plant growth and signalling, including attracting beneficial insects and triggering plant defences, or act directly to kill or inhibit plant pathogens or insects pests (Köhl et al. 2019; Mendoza‐Mendoza et al. 2024; Morath et al. 2012; Soto et al. 2021; Weisskopf et al. 2021). Because of their distinctive chemical properties and function as signalling molecules, microbial VOCs can be leveraged for agricultural applications, notably contributing to crop protection (Annaz et al. 2024; Bezerra et al. 2021; Wenda‐Piesik 2011). Volatiles more broadly from other organisms such as plants can modify insect feeding, reproduction, olfactory behaviour, and host preference (Bruno et al. 2018; Dos Santos et al. 2016; Hegde et al. 2011; Singh et al. 2021), enabling eco‐friendly pest management strategies that exploit these compounds as targeted repellents or attractants (Fountain et al. 2017; Reisenman et al. 2016; Wyatt 2018).

Previously, many microbial VOCs were considered mere metabolic by‐products, but recent research has overturned this notion. Compounds once dismissed as waste gases, such as acetoin, 2,3‐butanediol, or dimethyl disulfide, are now recognised as important mediators of plant growth promotion, plant defence activation, and microbial communication (Kim et al. 2013; Ryu et al. 2003; Tyc et al. 2017; Vespermann et al. 2007). Another example is the sulphur containing volatile S‐methyl methane thiosulfonate produced by bacteria isolated from the microbiome of potato plants, which showed strong in planta protection against late blight causing agent *Phytophthora infestans* (Chinchilla et al. 2019). These findings underscore how microbial metabolism and signalling are more chemically intertwined than previously thought, expanding the scope of what qualifies as ‘functional’ volatiles in ecology and agriculture.

Despite the growing body of research on microbial VOCs, translation of and evidence for these compounds in agricultural applications remains limited. Most studies are conducted in laboratories or under controlled conditions, yielding thousands of identified microbially produced VOCs, though only a small proportion (~10%) have an assigned biological function (Duc et al. 2022; Effmert et al. 2012; Kemmler et al. 2024). Recent reviews and compilations of case studies suggest that over 70% of published microbial VOC studies are limited to in vitro systems, while greenhouse or pot experiments make up about 20%–30%, and less than 5% involve open‐field evaluations (Montejano‐Ramírez et al. 2024; Rani et al. 2023; Schmidt et al. 2015; Schulz‐Bohm et al. 2017). Several articles have reviewed the latest research developments on microbial VOCs (Ali et al. 2025; Garbeva and Weisskopf 2020; Ledford et al. 2025; Poveda 2021; Razo‐Belman and Ozuna 2023; Salinas‐García et al. 2025). In this paper, we discuss the challenges of bridging the gap from discovery to application of microbial VOCs for crop protection. We explore how leveraging historical knowledge and recent findings on the ecological roles of microbial VOCs, along with understanding the limitations of translating laboratory results to the field, can help identify areas for future research focus and potential.

## 2 From Petri Dish to Field: Shifts in VOC Behaviour and Associated Challenges

Much of what we currently know about VOCs in plant protection stems from in vitro experiments, typically performed in sealed petri dishes or vials, where microbial cultures or plant tissues are exposed to concentrated volatile blends under highly controlled conditions (Fincheira and Quiroz 2018; Kanchiswamy et al. 2015; Piechulla and Degenhardt 2014). These systems have proven invaluable for identifying bioactive compounds and screening microbial strains with potential antifungal, antibacterial, or insect‐repelling activity. However, the artificial nature of these conditions, such as enclosed spaces, high nutrient media, and uniform VOC exposure, creates an environment that rarely reflects the complex, dynamic realities of agricultural settings (Razo‐Belman and Ozuna 2023). Current screening assays poorly capture the complexity of plant–soil–microbe interactions. Standard plate‐based systems overlook fluctuating oxygen levels, root exudates, soil structure and microbial diversity, all of which influence VOC diffusion and activity (Tilocca et al. 2020). An example of the importance of in situ testing is a recent study showing bacteria that emitted antifungal VOCs when grown on potato leaf surfaces, underscoring the necessity of evaluating these emissions under greenhouse and field conditions (Gfeller et al. 2022).

VOCs can influence biology and crop productivity at paddock scale. A well‐known example is the truffle *brûlé*, the bare zone around certain truffle‐producing trees, which is partly driven by fungal volatile emissions creating a competitive exclusion zone to outcompete the growth of microbes (Splivallo et al. 2011). This case demonstrates how field observations can be linked to specific VOCs through chemical analysis, while in many systems volatiles are characterised before their ecological functions are understood. Bridging this gap requires research in more realistic environments, as the in vitro–in vivo gap becomes apparent when VOCs transition from petri dishes to greenhouse or soil systems. Factors such as diffusion, adsorption, and microbial competition significantly reduce VOC availability and persistence in soils (Piechulla et al. 2017; Wheatley 2002). Soils act as reactive matrices that can bind or degrade volatiles based on moisture, temperature and pH, while rhizosphere microbes may metabolise VOCs before they reach targets. Consequently, even potent VOCs identified in the lab may have minimal impact in the field.

Field detection of VOCs presents significant analytical challenges. Rapid atmospheric dispersion and adsorption to soil micropores often reduce VOC concentrations below detection limits, while the wide variation in boiling points and water solubility complicates the interpretation of measured levels (Riu et al. 2022). Analytical constraints also limit VOC detection, as many are unstable or produced in trace amounts, thus falling below limits of detection of routine methods, or possess unusual chemical features that complicate identification (Effmert et al. 2012; Schulz‐Bohm et al. 2017). Innovative approaches, such as non‐invasive pathogen detection (Cagliero et al. 2021; Cui et al. 2018; Kim et al. 2024) and in situ soil profiling using Solid Phase Microextraction (SPME) (Deasy et al. 2016), are promising, but they still struggle due to the spatial and chemical complexity of soils. Nevertheless, when measurable, VOCs provide a valuable real‐time window into microbial activity in situ (Jung et al. 2023; Riu et al. 2022).

Environmental factors significantly complicate the translation of VOC production from lab to field conditions. Microbes produce different blends of VOCs based on substrate and nutrient availability; for example, *Fusarium* emits a more diverse range of VOCs on potato sucrose agar than on wheat kernels (Savelieva et al. 2016). Compounds identified as antifungal in vitro, such as 2,4,6‐trimethylpyridine in labs/closed vessels (Belt et al. 2025), still require validation in open environments. Closed systems often generate unrealistically high VOC concentrations, which can amplify biological responses and raise questions about cost‐effective field‐scale delivery. In many cases, VOC‐producing inoculants may be more feasible than direct application.

Finally, field application remains challenging and is influenced by soil type, climate, and microbial community context. Many VOCs are lipophilic and poorly soluble in water, limiting practical delivery (Garbeva and Weisskopf 2020). Consequently, active research is focused on formulations that maintain effective concentrations while minimising losses, including encapsulation and nanotechnologies (An et al. 2022; Zaman et al. 2025), biochar carriers, and microbes engineered or selected for sustained in situ production (Das et al. 2025; Fadiji et al. 2024). Ultimately, effective deployment depends on production, formulation, dosage, delivery method, and an understanding of how VOCs move and interact within environmental matrices (Ledford et al. 2025).

## 3 Ecology‐Based Design: Mimicking Nature for VOC Development and Deployment

In natural ecosystems, microbial VOC production is context‐dependent, mainly shaped by microbial community dynamics, plant species, environmental conditions and other biotic stressors. This complexity and the vast chemical diversity of VOCs present both challenges and opportunities for agricultural innovation. Bridging the in vitro–in vivo divide requires rethinking how microbial VOCs are discovered, characterised and tested. Future efforts, as outlined in Box 1 and the following case studies, should integrate an understanding of the ecological context of VOCs, including knowledge from plant‐microbe interactions, inter‐species and inter‐domain microbial community interactions, as well as diverse soil and field conditions. This approach will enable researchers to assess how producer strains and their VOCs function in agricultural environments, ultimately helping to protect crops and produce from pests and diseases.

BOX 1 **Exploiting the ecological role of microbial VOCs for agricultural application in crop protection.**

**Table:** 

Category	Ecological role of VOCs	Exploitation in crop protection
Function	Microbial communication, attraction or dispersal of insects, and protection of the host plant	Direct pathogen or pest suppression, attraction of beneficial organisms, priming of crop defence and promotion of crop growth
Source	Naturally emitted by microbes	Microbial inoculant, microbial synthetic community, purified microbial VOCs and synthetic VOCs
Target organisms	Soil microbes, endophytes and beneficial insects	Pests, pathogens, beneficial insects, and crops, with limited off‐target effects
Spatio‐temporal dynamics	Dynamics and context‐dependent, influenced by stress and environmental factors	Application aligned with pathogen or pest life‐cycle, crop phenology and the timing and conditions of produce in storage
Application method	Passive emission, no human intervention	Controlled release systems, including irrigation, soil drench, seed, foliar spray, and biofumigation

### 3.1 Insights From Plant VOCs in Agriculture and Strategies for Using Natural or Purified Volatiles

Although microbial‐derived VOCs for crop protection are still in early stages of development, examples from plant‐derived volatiles demonstrate their potential. *Brassicaceae* residues illustrate how VOCs can be harnessed at scale: glucosinolates in *brassica* tissues decompose into sulfur‐containing volatiles, including isothiocyanates (ITCs), with antifungal and nematocidal activity (Pavana Praneetha et al. 2025). Incorporating this biomass into soil, known as biofumigation, is now widely used for pathogen control. Notably, glucosinolate catabolism is catalysed by both plant and microbial enzymes (Ji et al. 2024), suggesting opportunities to pair *brassica* biofumigation with microbial VOC producers. Biofumigation serves as a clear example of a VOC‐based strategy that has successfully progressed from laboratory study to field application.

Leveraging microbes already present in agricultural soils can be a more practical and cost‐effective strategy than applying inoculants or purified VOCs. Disease‐suppressive soils, shaped by distinctive microbial communities and soil properties, can naturally limit soil‐borne pathogens even in the presence of a susceptible host (Schlatter et al. 2017; Wen et al. 2023). This suppression can involve sulphurous VOCs produced by bacterial strains from the *Burkholderiaceae* family for example (Carrión et al. 2018). Ossowicki et al. (2020) also identified volatile‐mediated suppression in both disease suppressive and conducive soils, indicating that while VOCs may contribute, they are not solely responsible for the disease suppression activity (Ossowicki et al. 2020). A promising strategy to increase or diversify microbial VOC production is to add specific organic substrates that act as volatile precursors (Garbeva and Weisskopf 2020), thereby enhancing the targeted metabolic pathways. This could be further enhanced by targeting disease suppressive VOCs common across a range of microbes and plants such as tropone and related compounds (Duan et al. 2020; Gallart et al. 2025).

Alternatively, several experimental studies have successfully delivered purified VOCs or VOCs from microbial culture filtrates into soils via drip irrigation or injection to control soil pests or diseases or improve yield under field conditions (Baroja‐Fernández et al. 2021; Pecchia et al. 2017; Yan et al. 2019). This includes the commercial use of dimethyl disulfide (DMDS) as a soil fumigant (Pecchia et al. 2017). Dimethyl disulfide is marketed as Paladin in several countries and is sold as a liquid pre‐plant soil fumigant for the control of soil‐borne pests and diseases.

### 3.2 Harnessing Microbe‐Microbe Interactions With Synthetic Microbial Communities

Synthetic microbial communities or SynCom represent an exciting new approach to harnessing the ecological properties of VOCs in microbial communication to elicit novel VOC production. For example, in a study led by Lee et al. (2025) conducted under both greenhouse and field conditions, a SynCom of four gram‐positive bacteria significantly reduced aphid infestation in pepper plants. This effect was attributed to the activation of plant defence gene expression and the coordinated production of the VOC 1‐nonanol, which could not be achieved by any single strain within the SynCom alone. Similarly, Türksoy et al. (2025) showed a root‐derived 16‐strain bacterial SynCom produced a distinct VOC profile that differed from the sum of the VOCs produced by its individual members, with not all compounds emitted by the individual strains present in the community VOC mixture. Although the community was assembled using equal proportions of all 16 strains, it quickly became dominated by three strains. This suggests the community VOC profile may be governed by a few strains, which has implications for the field delivery of SynComs into complex soil environments.

### 3.3 Amplifying the Response

VOCs typically act within a limited spatial and temporal range, but their ecological ripple effects can extend protection beyond the immediate release zone. Some VOCs induce systemic resistance or prime plant defences, safeguarding both treated and distal tissues (Belt et al. 2021; Brosset and Blande 2022; Lee et al. 2012). Others influence higher trophic levels: for example, soil application of 3‐pentanol induced defence gene expression and reduced *Pseudomonas syringae* disease in cucumber plants while attracting predatory ladybird beetles that suppressed aphids (Song and Ryu 2013). Certain volatiles from the *Streptomyces* genus, such as 2‐methylisoborneol, attract insects to oviposit and subsequently kill larvae via co‐produced metabolites (Ho et al. 2020). Similarly, geosmin acts as a warning cue to deter nematode predation (Garbeva et al. 2023; Zaroubi et al. 2022), a mechanism that is now being commercially exploited through *Streptomyces*‐based seed treatments that create a protective zone around roots (Indigo Ag). Conversely, geosmin, as a strong attractant for certain mosquito species (Garbeva et al. 2023), could be exploited in mosquito control by using geosmin to bait traps and increase capture rates.

### 3.4 Dual‐Purpose VOCs – Expanding Beyond Pesticidal Roles With Biostimulants

Beyond their pesticidal functions, many microbial VOCs have emerged as potent biostimulants that promote plant growth and resilience. Volatiles have been shown to stimulate root elongation, lateral root formation and nutrient uptake by modulating hormonal signalling pathways, including auxin and cytokinin responses (Jahan et al. 2024; Ryu et al. 2003). These plant growth–promoting volatiles act as airborne signals, allowing beneficial microbes in the rhizosphere to influence host physiology without direct contact. This phenomenon challenges the traditional distinction between biopesticides and biostimulants, suggesting that microbial volatiles can simultaneously enhance plant performance while maintaining protective functions (Garbeva and Weisskopf 2020; Pantigoso et al. 2022). A recent study demonstrated that *Paenibacillus peoriae* emits VOCs that significantly enhance the growth of *Arabidopsis* plants by stimulating photosynthesis‐related pathways, while the same species is also known for producing antimicrobial metabolites, underscoring its potential as a dual‐function plant‐beneficial microbe (Wang, Lin, et al. 2023).

In addition to promoting growth, VOCs can also enhance stress tolerance, helping plants withstand abiotic constraints such as drought, salinity and oxidative stress (Song et al. 2021). Volatiles emitted by various microbial species have been shown to induce systemic tolerance through the activation of antioxidant enzymes, osmolyte accumulation and modulation of stress‐responsive genes (Cellini et al. 2021; Jahan et al. 2024; Singh et al. 2025; Wadduwage et al. 2024). These mechanisms overlap with defense priming against pathogens, highlighting the dual‐function nature of VOCs as both protective and stimulatory agents.

### 3.5 Post‐Harvest Applications

An area where the characteristics of VOCs show promise in crop protection is in post‐harvest disease control, or biofumigation of stored fruits, vegetables and grains, where the concentration and dispersion of VOCs can be effectively controlled. This is particularly relevant for produce storage where resistance to chemical controls has developed, or where no post‐harvest chemical controls exist due to food safety and strict residue requirements. Microbial VOCs have been widely studied for their potential in controlling post‐harvest diseases, with many examples demonstrating strong antimicrobial effects against post‐harvest pathogens, thereby reducing spoilage and improving produce quality (Razo‐Belman and Ozuna 2023). This includes tubers, fruits, seeds, and nuts (Boukaew and Prasertsan 2020; Elsherbiny et al. 2020; Santra and Banerjee 2023; Saxena and Strobel 2021; Strobel et al. 2001).

One well‐studied microbial VOC with potential commercialization is derived from fungal strains of the *Muscodor* genus (Saxena and Strobel 2021). For example, the colonisation of sterile dried rye grain with *M. albus* is effective at controlling decay and spoilage in apples caused by blue mould (*Penicillium expansum*) and grey mould (*Botrytis cinerea*), attributed to production of isobutyric acid and 2‐methyl‐1‐butanol (Mercier and Jiménez 2004). Volatile‐generating sachets of *M. albus* colonised rye grain have been developed and are effective in controlling decay of table grapes under commercial conditions (Mercier et al. 2010). In this example, a continuous production of VOCs is achieved; however, it may also be possible to develop substrates or membranes for slow release of VOCs once these are imbibed.

### 3.6 From Discovery to Application: Technology Transfer of VOC‐Based Solutions

For VOC‐based crop protection to move beyond proof‐of‐concept, major effort is needed in technology transfer, including scalable production, stabilisation, formulation and delivery. Unlike conventional pesticides, VOCs are often short‐lived and prone to rapid diffusion, which makes both storage and controlled field delivery challenging. One option is mass production of VOC‐producing microbes using standard industrial fermentation systems, similar to those used for biofertilisers or biopesticides (Bashan et al. 2014; Malusá et al. 2012). Here, selection of high‐VOC‐yield strains, optimisation of carbon and sulphur precursors, and control of aeration and pH can strongly influence volatile output (Effmert et al. 2012; Garbeva and Weisskopf 2020; Kai et al. 2007, 2009). Co‐fermentation or staged feeding of specific substrates offers a way to bias production toward desired volatiles rather than broad, uncontrolled mixtures (Bode and Müller 2005; Demain and Fang 2000; Jalil and Yu 2024; Perez‐Esteban et al. 2022).

Formulation is equally critical. For living inoculants, encapsulation in granules, pellets, or polymer matrices can protect microbes during storage and release them slowly into soil, prolonging VOC production in situ (Fadiji et al. 2024; Vassilev et al. 2020). For non‐living products, controlled‐release technologies, such as microencapsulation, adsorption onto biochar, clays or organic carriers, or inclusion in oil‐based emulsions, can reduce volatilisation losses and extend biological activity (Jasrotia et al. 2022; Kaikiti et al. 2021; Werdin González et al. 2014; Zatta et al. 2023).

Delivery methodology determines whether VOCs can realistically be applied on‐farm. As discussed earlier, soil‐targeted approaches including drip irrigation systems and injection have been successfully demonstrated. Other methods include seed coatings using polymers or incorporation into fertiliser or organic amendment granules (Marín‐Martínez et al. 2021; Singh et al. 2023; Srivastava et al. 2024). Importantly, delivery systems must align with existing farm machinery and practices to minimise adoption barriers. The commercial use of dimethyl disulfide as a liquid fumigant illustrates that with appropriate formulation and application technology, even highly volatile compounds can be deployed at scale (Pecchia et al. 2017; Yan et al. 2019).

Finally, regulatory approval, safety and farmer acceptance are central to technology transfer. VOC‐based products must demonstrate consistent efficacy across environments, low off‐target toxicity and manageable odour and handling properties. Integration with precision‐agriculture tools, such as variable‐rate application or soil microbiome diagnostics, could allow VOC strategies to be tailoured to specific fields and disease risks.

## 4 Limitations and Outlook

**FIGURE 1:**
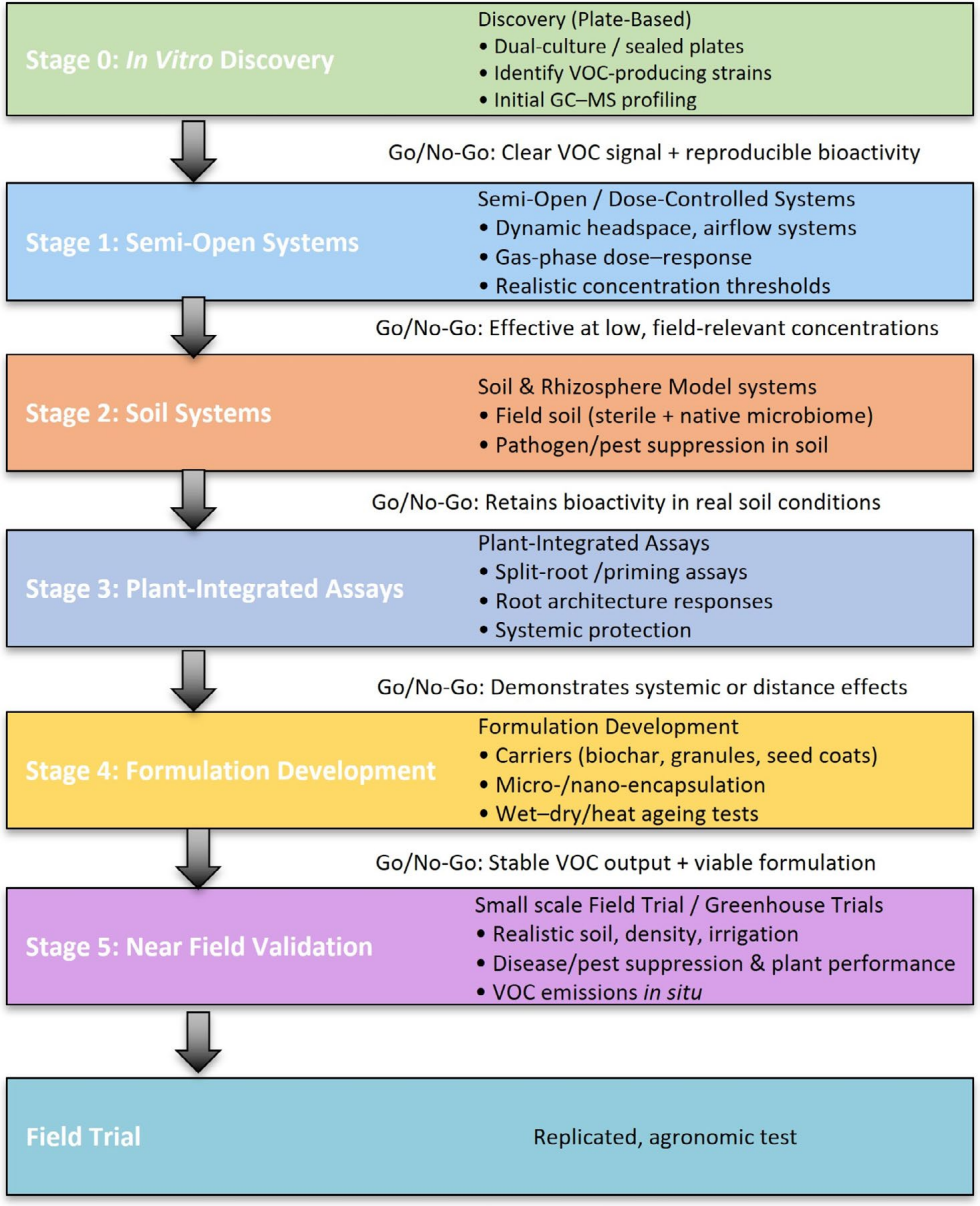
Suggested workflow of VOC screening where research progresses from current in vitro discoveries (Stage 0) or moves immediately to semi‐open systems (Stage 1) more realistic of field conditions.

Ultimately, microbial VOCs offer significant potential for crop protection. However, success hinges on our ability to translate complexity into practicality, bridging the gap between the lab bench and the farm gate. Translating ecological insights into practical applications represents a pathway to achieve this. Five years ago, Garbeva and Weisskopf (2020) highlighted several fundamental questions that needed addressing to advance our knowledge of VOCs and their interactions with plants. In the context of crop protection, key questions included ‘What are the key VOCs that are crucial for plant health, and by which microbes are they emitted?’, ‘What are the modes of action underlying the health‐protecting effects of microbial VOCs?’, ‘Under which ecologically relevant conditions do microbes produce plant health‐protecting VOCs?’, ‘What is the spatial scale of VOC‐mediated plant–microbe interactions?’, ‘What is the best way of applying VOCs or VOC‐emitting strains?’ and ‘How can we steer/stimulate plant microbiota to emit beneficial VOCs?’ While many of these remain poorly answered, we highlighted exciting progress in understanding ecologically relevant conditions for microbial VOC production and impacts on plant health. In particular, insights from synthetic microbial community studies, and new knowledge on dual‐purpose crop protection VOCs such as biostimulants, insect attractants and warning signals, as well as ground‐truthing studies through field‐validation and product commercialization have emerged. However, we have identified the following knowledge gaps that continue to limit the translation of ecological functions into field application:

1. Lack of in‐depth knowledge of microbe‐microbe interactions. Most knowledge of microbial VOCs is derived from laboratory studies on single strains, which do not consider interactions with other microbes in the soil, rhizosphere or phyllosphere, or with plant hosts.
2. Need for improved in vivo screening approaches that simulate soil, rhizosphere and in planta conditions. It is essential to integrate plants early in the screening process by utilising for example, rhizobox or split‐root systems. Dose–response experiments should be conducted under realistic conditions in soil or plant systems, rather than solely on agar plates (Figure 1). This also extends to field testing frameworks that encompass multiple seasons and multiple sites, including both in‐crop and post‐harvest trials, while engaging growers early in the evaluation process to understand the practicality, cost‐effectiveness and integration of VOCs with existing practices.
3. Assessing non‐target effects early to avoid unintended consequences. Broad‐spectrum VOCs may impact non‐target organisms, including beneficial insects or soil microbes, or impact food or worker safety.

Deciding whether to apply VOCs or their microbial producer strains will vary on a case‐by‐case basis, depending on crop, pest pressure, and environmental context. In some settings, an integrated approach that combines both strategies may be beneficial. It is noteworthy that synthetic products consisting of VOC formulations are rare. In contrast, many microbial strains that produce VOCs are already included in commercial biocontrol and biostimulant products. Therefore, microbial inoculants may offer a quicker impact pathway for VOCs, leveraging their broad ecological functions to deliver sustainable crop protection tools for farmers. Furthermore, advances in synthetic biology present new opportunities to design microbes or plants that can stably and predictably produce protective VOCs under field conditions. Engineered strains could optimise VOC output, enhance resilience against environmental fluctuations, or couple volatile production to specific plant or pathogen cues, creating more targeted and reliable biocontrol strategies (Aminian‐Dehkordi et al. 2023). At the same time, digital and sensor technologies are being developed to transform how VOCs are monitored and deployed. Emerging VOC biosensors and pathogen‐detection tools can provide real‐time insights into soil and crop health, while drone‐based sensing and distributed sensor networks enable spatial mapping of VOC patterns (Berger et al. 2022; Gan et al. 2023; MacDougall et al. 2022; Rezaee Danesh 2025). These systems could detect biologically relevant concentrations across field environments, guiding more precise and efficient application of VOC‐based solutions.

## 5 Conclusion

Microbial VOCs represent a powerful but underexploited resource for sustainable crop protection. While laboratory studies have revealed remarkable biological activities, real‐world impact depends on understanding how VOCs behave in complex field environments and designing systems that mimic their ecological functions. Future success will rely on advances in bioproduction, formulation science and delivery technology to transition VOC‐based approaches to crop protection from experimental systems to practical, on‐farm tools.

## Author Contributions

**Marta Gallart:** conceptualization, writing – original draft, writing – review and editing. **Katharina Belt:** conceptualization, writing – original draft, writing – review and editing. **Louise F. Thatcher:** conceptualization, writing – original draft, writing – review and editing. **Lachlan Dow:** conceptualization, writing – original draft, writing – review and editing.

## Funding

The authors have nothing to report.

## Conflicts of Interest

The authors declare no conflicts of interest.

## Data Availability

The authors have nothing to report.
